# Clinical and Kinematic Features of Valproate-Induced Tremor and Differences with Essential Tremor

**DOI:** 10.1007/s12311-020-01216-5

**Published:** 2020-11-16

**Authors:** Giulia Paparella, Luca Angelini, Alessandro De Biase, Antonio Cannavacciuolo, Donato Colella, Carlo Di Bonaventura, Anna Teresa Giallonardo, Alfredo Berardelli, Matteo Bologna

**Affiliations:** 1 IRCCS Neuromed, Pozzilli (IS), Italy; 2 Department of Human Neurosciences, Sapienza University of Rome, Viale dell’Università, 30, 00185 Rome, Italy

**Keywords:** Valproate-induced tremor, Essential tremor, Tremor classification, Kinematic, Neurophysiology, cerebellum

## Abstract

Tremor is a common movement disorder that can be induced by medications, including valproate, which is used for the treatment of epilepsy. However, the clinical and neurophysiological features of valproate-induced tremor are still under-investigated. We performed a clinical and kinematic assessment of valproate-induced tremor by considering tremor body distribution and activation conditions. We investigated possible correlations between demographic and clinical data and kinematic features. Valproate-induced tremor results were also compared with those collected in a large sample of patients with essential tremor. Sixteen valproate-induced tremor patients and 93 essential tremor patients were enrolled. All participants underwent a standardised neurological examination and video recording. Patients also underwent an objective assessment of postural, kinetic and rest tremor of the upper limbs and head tremor through kinematic analysis. Nonparametric tests were used for statistical comparisons between the two groups. Clinical evaluation showed a higher occurrence of rest tremor as well as head or voice, and lower limb involvement in patients with valproate-induced tremor. Kinematic analysis showed a substantial variability in the tremor features of patients with valproate-induced tremor. Compared to essential tremor, we found a higher occurrence of rest tremor of the upper limbs and the involvement of more body segments in valproate-induced tremor patients. Valproate-induced tremor has distinctive clinical and kinematic features, which may suggest that valproate interferes with the cerebellar functions.

**Supplementary Information:**

The online version contains supplementary material available at 10.1007/s12311-020-01216-5.

## Introduction

Tremor is an involuntary, rhythmic and oscillatory movement of a body part and is present in several neurological conditions [1]. Tremor can be caused by the use of several drugs [2], particularly anticonvulsants, like valproate. The specific time relationship with treatment initiation is crucial in the definition of valproate-induced tremor (VIT) [2–5]. Although VIT is relatively frequent [4], only a limited number of case reports and clinical studies have investigated its major features, indicating that VIT is mainly characterized by postural and kinetic tremor of the upper limbs [6–9]. Some major clinical features of VIT include dose-dependent severity [5], though no clear dosage threshold for VIT occurrence has been identified [5], and a relatively mild progression over time [2, 4].

A limited number of studies have characterised VIT from a neurophysiological point of view [5, 10–12]. Overall, these studies have indicated that VIT mainly consists of postural tremor of the upper limbs with a low amplitude and relatively high frequency (ranging from 6 to 15 Hz), though rest tremor of the upper limbs has also been confirmed in some patients [5, 10, 13, 14]. However, no previous neurophysiological studies on VIT have comprehensively assessed tremor by considering activation conditions (postural, kinetic and rest tremor) and body distribution, as proposed by the new consensus statement [1]. In addition, no studies have considered possible correlations between VIT clinical and neurophysiological features.

In the present study, we aimed to better characterise clinical and kinematic features of VIT using standardized clinical scales and an objective assessment of tremor through motion analysis [15–17]. To this aim, we investigated postural, kinetic and rest tremor in different body parts, including the upper limbs and head [15–17]. We investigated possible correlations between demographic, clinical and kinematic data in VIT patients. Like VIT, essential tremor (ET) is a common movement disorder characterized by postural and kinetic tremor of the upper limbs [1]. ET is clinically heterogeneous, and its diagnosis is often challenging since differences in clinical and neurophysiological features between VIT and ET have been scarcely investigated [14]. A comparison study may help in the differential diagnosis between VIT and ET and may provide further insight into the pathophysiological mechanisms of both tremor types. Therefore, we compared a group of VIT patients with a group of ET in order to identify specific elements to better characterize VIT and provide a deeper understanding of tremor pathophysiology in this condition.

## Methods

### Participants

We enrolled 16 VIT patients, consecutively recruited from the outpatient clinic of the Department of Human Neurosciences, Sapienza University of Rome, from February 2018 to December 2019. VIT patients had a diagnosis of epilepsy according to International League Against Epilepsy (ILAE) criteria [18]. All VIT patients were under valproate therapy for at least 2 years prior to study enrolment. In all cases, tremor onset started after valproate administration, with no prior tremor manifestations. No patients had a history of other neurological conditions characterized by tremor and none was taking any other medication for the treatment of tremor [2]. Detailed clinical information is presented in Tables 1 and 2. Four VIT patients were in monotherapy with valproate and 12 were taking other anticonvulsants in addition to valproate (Table 2).

**Table 1:** Demographic and clinical data of VIT and ET patients

	VIT (16)	ET (93)	*P*
Sex	7F/9M	39F/54M	0.39
Age (years)	45.19 ± 11.42	68.5 ± 10.89	*< 0.01*
MOCA	23.29 ± 4.43	25.58 ± 2.35	0.09
FAB	14.92 ± 2.47	15.87 ± 1.82	0.23
BAI	16.67 ± 18.23	15.5 ± 16.35	0.53
BDI-II	15.46 ± 15.58	14.86 ± 15.92	0.62
Valproate treatment duration	15.06 ± 13.16	-	
Valproate mean dosage/die (mg)	1331.25 ± 368.27	-	
Valproate serum concentration (μg/ml)*	82.19 ± 15.08	-	
Age at tremor onset (years)	37.25 ± 17.20	55.47 ± 17.06	*< 0.01*
Tremor duration (years)	8.88 ± 8.46	13.03 ± 13.12	0.17
Familial history	3Y/13 N	50Y/43 N	*0.01*
Tremor clinical data scores			
FTMTRS total	27.63 ± 12.23	19.47 ± 12.46	*0.01*
FTMTRS section A	10.63 ± 6	6.84 ± 4.14	*< 0.01*
FTMTRS section B	10.80 ± 4.75	7.90 ± 5.81	*0.03*
FTMTRS section C	6.81 ± 4	3.86 ± 3.49	*< 0.01*
Head and/or voice tremor (no. of patients)	11Y/5 N (68.75%)	30Y/63 N (32.25%)	*< 0.01*
Lower limb tremor (no. of patients)	8Y/8 N (50%)	6Y/87 N (6.25%)	*< 0.01*
Rest tremor (no. of patients)	9Y/7 N (56.25%)	10Y/83 N (10.75%)	*< 0.01*

*BAI* Beck Anxiety Inventory, *BDI-II* Beck Depression Inventory, *ET* essential tremor, *F* female, *FAB* Frontal Assessment Battery, *FTMTRS* Fahn-Tolosa-Marin Tremor Rating Scale, *M* male, *MOCA* Montreal Cognitive Assessment, *UL* upper limb, *VIT* valproate-induced tremor. All values are expressed as average ± standard deviation. Significant values are in italics. *Valproate serum concentration (μg/ml) was measured in 11 patients

**Table 2:** Diagnosis and antiepileptic therapy in patients with VIT

	Diagnosis	Treatment and daily dose
1	Focal epilepsy (unknown aetiology)	VPA 1000 mg; LTG 300 mg; ZNS 200 mg
2	Idiopathic generalized epilepsy	VPA 1000 mg
3	Focal epilepsy (unknown aetiology)	VPA 1000 mg
4	Idiopathic generalized epilepsy	VPA 1500 mg; LTG 200 mg
5	Idiopathic generalized epilepsy	VPA 1500 mg; LTG 150 mg
6	Idiopathic generalized epilepsy	VPA 1500 mg; ETS 1250 mg; FNB 25 mg
7	Idiopathic generalized epilepsy	VPA 1500 mg; LTG 200 mg
8	Focal epilepsy (structural origin)	VPA 2000 mg; LEV 3000 mg; LTG 300 mg
9	Idiopathic generalized epilepsy	VPA 1000 mg; LTG 350 mg; TPM 200 mg
10	Focal epilepsy (structural origin)	VPA 1000 mg; LCS 300 mg; LEV 2000 mg
11	Idiopathic generalized epilepsy	VPA 2000 mg; LTG 150 mg; RFM 3600 mg
12	Idiopathic generalized epilepsy	VPA 1500 mg
13	Idiopathic generalized epilepsy	VPA 1500 mg; LTG 150 mg
14	Idiopathic generalized epilepsy	VPA 800 mg
15	Idiopathic generalized epilepsy	VPA 1500 mg, LEV 2000 mg; PER 10 mg
16	Focal epilepsy (structural origin)	VPA 1000 mg; CBZ 20 mg; LCS 200 mg; LEV 2000 mg

*CBZ* clonazepam, *ETS* ethosuximide, *FNB* phenobarbital, *LCS* lacosamide, *LEV* levetiracetam, *LTG* lamotrigine, *PER* perampanel, *RFM* rufinamide, *ZNS* zonisamide, *VIT* valproate-induced tremor, *VPA* valproate

We also consecutively recruited 93 patients with ET who were diagnosed according to the most recent clinical criteria [1] from our Department’s outpatient clinic from February 2018 to December 2019 (Table 1). All tremor treatment in the ET group was discontinued 48 h before assessment.

All participants provided written informed consent to the study. Experimental procedures were approved by the local ethics committee and performed according to the Declaration of Helsinki.

### Clinical Evaluation

Demographic and clinical data collection included sex, age, comorbidities, familial history, tremor onset and duration and concomitant antiepileptic treatment (Tables 1 and 2). Possible cognitive and psychiatric disturbances were evaluated by means of the Montreal Cognitive Assessment (MOCA), Frontal Assessment Battery (FAB), Beck Anxiety Inventory (BAI) and Beck Depression Inventory (BDI-II).

All participants underwent a neurological examination performed by two blinded neurologists expert in movement disorders. Tremor was clinically assessed using the Fahn-Tolosa-Marin Tremor Rating Scale (FTMTRS) [19].

### Kinematic Recordings and Analysis

Kinematic recordings were performed using an optoelectronic system (SMART motion system, BTS Engineering, Italy) consisting of three infrared cameras (sampling rate of 120 Hz) and reflective markers of negligible weight taped to the subject’s arms, trunk and head [15–17]. For the upper limbs, we used four markers placed on the distal phalange of the index finger and on the second metacarpal bone of each hand. To derive the head coordinate system, we used three reflective markers: two placed over the frontal orbital processes (bilaterally) and one placed over the nasion. Three markers were also placed on the trunk to define a reference plane that allowed possible contamination due to trunk movements to be excluded from the upper arm and head movement recordings [15–17]. Upper limb postural tremor was recorded: (i) with the arms outstretched in front of the chest (posture 1), and (ii) with the arms flexed at the elbows i.e. lateral ‘wing beating’ posture (posture 2). Three 45-s recordings were obtained for each posture. Upper limb kinetic tremor was recorded in three 15-s recording blocks during a ‘pointing task’, in which subjects were asked to repetitively move their index finger from their nose to a reflective target fixed on a heavy support approximately 15 cm above the table at sternal height and at approximately 2/3 arm distance [15–17]. Rest tremor of the upper limbs and head was recorded while patients sat comfortably on a chair facing the cameras, with their arms lying on a desk in front of them. Three 45-s recordings per patient were collected [15–17].

Tremor analysis was performed using dedicated software (SMART Analyzer, BTS Engineering, Italy). For postural and rest tremor analysis, the marker placed on the second metacarpal bone was considered the reference marker, as in previous studies [15–17]. We determined the magnitude of postural and rest tremor by measuring the root mean square (RMS) of the acceleration traces of the reference marker in 3D space (GRMS^2). Power spectra were calculated by means of fast Fourier transformation. We then measured the dominant frequency peak (Hz) of postural and rest tremor. For kinetic tremor analysis, the marker placed on the last phalange of the index finger was considered the reference marker. Kinetic tremor of the upper limbs was measured as previously detailed [15–17]. We considered the number of movements and the distance (m) covered by the arm endpoint during the ‘pointing task’. As measures of movement execution, we considered velocity peak (m/s) and acceleration peak (m/s^2^). The duration of the acceleration and deceleration phases, the deceleration/acceleration ratio (D/A) and the curvature index (CI, i.e. arm endpoint average path length/length of a straight line joining the initial and final positions) served as measures of movement quality, i.e. trajectory homogeneity [15–17].

### Statistical Analysis

Mann-Whitney *U* test was used to assess age differences between groups. Sex differences and other categorical variables were expressed as frequencies, and compared using the chi-square test. Numerical data were expressed as mean values ± 1 standard deviation (SD) unless otherwise specified and compared using Mann-Whitney *U* test. In a preliminary analysis, we first considered data from the right and left side of each upper limb kinematic parameter of postural, rest and kinetic tremor in the two groups. We then computed an asymmetry index (more affected side − less affected side) / (more affected side + less affected side). The asymmetry indices of amplitude and frequency values recorded in VIT and ET during posture 1, posture 2 and the rest position were compared with two separate repeated measures analyses of variance (rmANOVAs) with the factors GROUP (two levels: VIT vs. ET) and POSTURE (three levels: posture 1, posture 2 and rest). Asymmetry indices for the kinematic parameters of the pointing task were compared with multivariate ANOVA (MANOVA). No differences between groups emerged in terms of tremor asymmetry (see Supplementary Tables 1 and 2). We thus computed the average of right and left side tremor magnitude (GRMS^2) and frequency (Hz) values of upper limb postural tremor in both groups for within (VIT or ET posture 1 vs. VIT or ET posture 2) and between (VIT posture 1 or 2 vs. ET posture 1 or 2) group comparisons. Data were then compared using Wilcoxon signed-rank test and Mann-Whitney *U* test. Mann-Whitney *U* test was also used to compare the average from the right and left side of the kinetic tremor parameters between VIT and ET patients. Possible relationships between clinical and neurophysiological data were assessed using Spearman correlations. All results are presented as mean values ± 1 SD unless otherwise specified. The level of significance was set at *p* < 0.05. Results were corrected for multiple comparisons using false discovery rate (FDR). Data were analysed using STATISTICA® (TIBCO Software Inc., Palo Alto, California, USA).

## Results

### Clinical and Kinematic Features of VIT

**Fig. 1:**
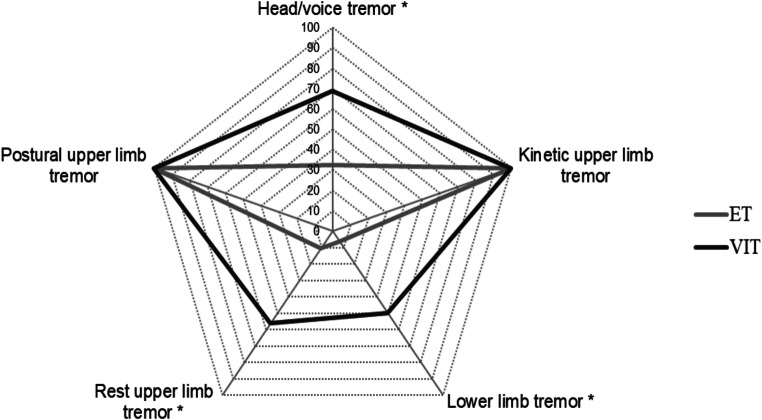
Comparison of tremor frequency (%) in different body parts in patients with valproate-induced tremor (VIT) (dark grey) and essential tremor (ET) (light grey). Asterisks indicate *p* < 0.05 with chi-square test

Clinical evaluation showed that all VIT patients had upper limb postural and kinetic tremor (100%), while upper limb rest tremor was clinically present in 9 out of 16 VIT patients (56.25%). Head tremor was present in 7 VIT (43.75%), voice tremor was observed in 9 VIT (56.25%) and lower limb tremor was observed in 8 out of 16 VIT patients (50%) (Table 1 and Fig. 1). VIT tremor severity as assessed by FTMTRS total score and FTMTRS subsections is detailed in Table 1.

**Fig. 2:**
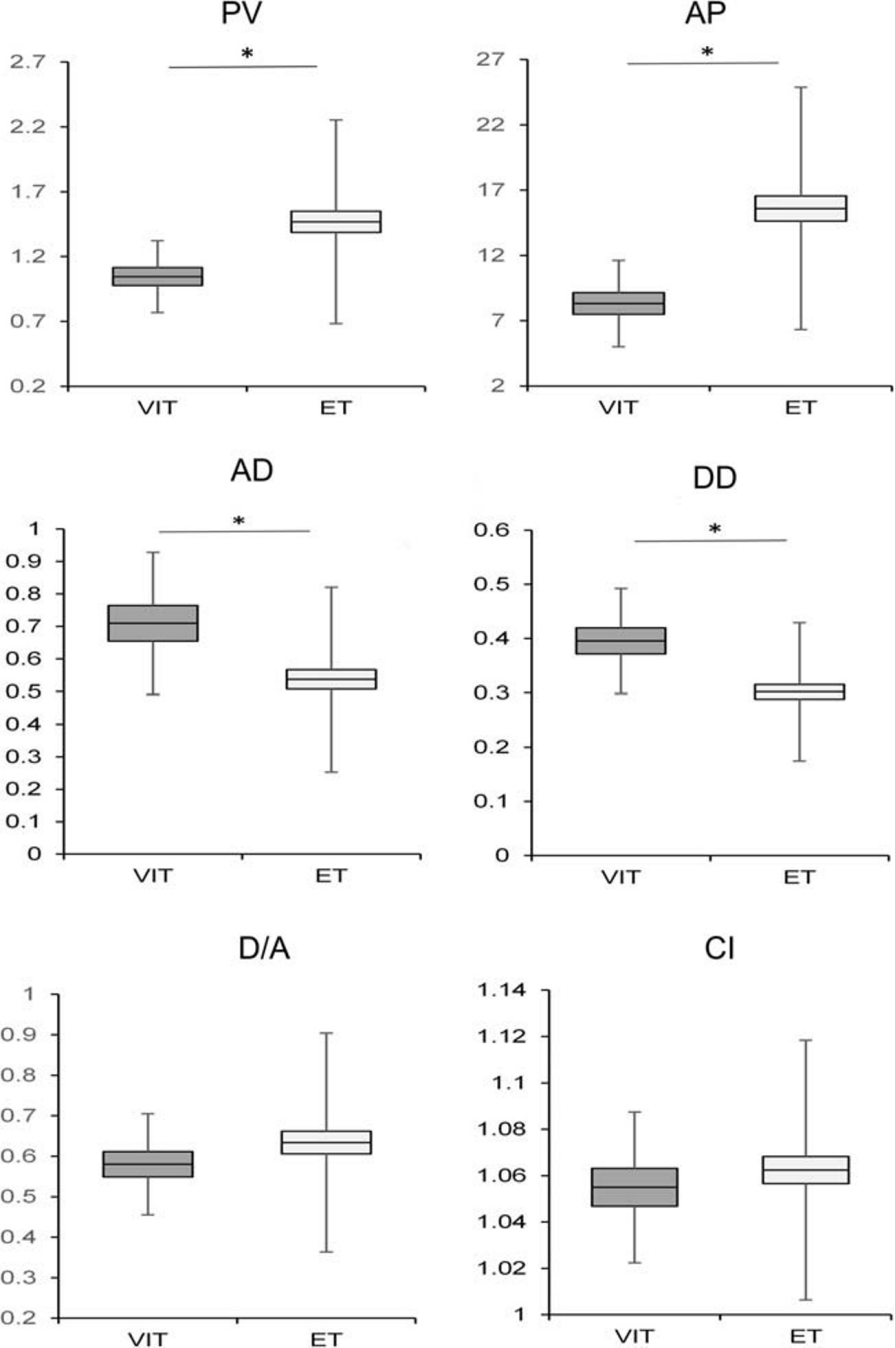
Kinematic parameters obtained of the pointing task for the kinetic tremor assessment in patients with valproate-induced tremor (VIT) (dark grey) and essential tremor (ET) (light grey). AD, acceleration duration, expressed in min; AP, acceleration peak, expressed in m/s^2^; CI, curvature index; D/A, deceleration/acceleration ratio; DD, acceleration duration, expressed in min; PV, peak of velocity, expressed in m/s. Horizontal lines denote average values. Boxes contain the mean value ± 1 standard error of the mean. Whiskers contain the mean value ± 1 standard deviation of the mean. Asterisks indicate *p* < 0.05 in post hoc comparisons

Kinematic analysis showed a substantial variability within the VIT group in terms of postural tremor amplitude (mean ± SD: 0.47 ± 0.44 GRMS^2 during posture 1; 0.92 ± 1.48 GRMS^2 during posture 2) and, to a lesser extent, frequency (mean ± SD: 4.46 ± 1.64 Hz during posture 1; 5.96 ± 1.28 Hz during posture 2) (Table 3). Amplitude and frequency of rest tremor of the upper limbs and head tremor were also variable. Patient variability in kinetic tremor was also reflected in the kinematic data obtained during the pointing task (Fig. 2).

**Table 3:** Kinematic data of postural upper limb tremor in VIT and ET patients

	GRMS^2			Hz		
	P1	P2	*p* values*	P1	P2	*p* values*
VIT	0.47 ± 0.44	0.92 ± 1.48	0.7057	4.46 ± 1.64	5.96 ± 1.28	*0.0063*
ET	0.34 ± 0.33	0.90 ± 2.12	*0.0001*	4.78 ± 1.75	5.32 ± 0.96	*0.0041*
*p* values**	0.4608	0.4484		0.6152	0.4484	

Significant values are in italics

*ET* essential tremor, *P1* posture 1, *P2* posture 2, *VIT* valproate-induced tremor. *P values with Wilcoxon signed-rank test; **P values with Mann-Whitney *U* test

**Fig. 3:**
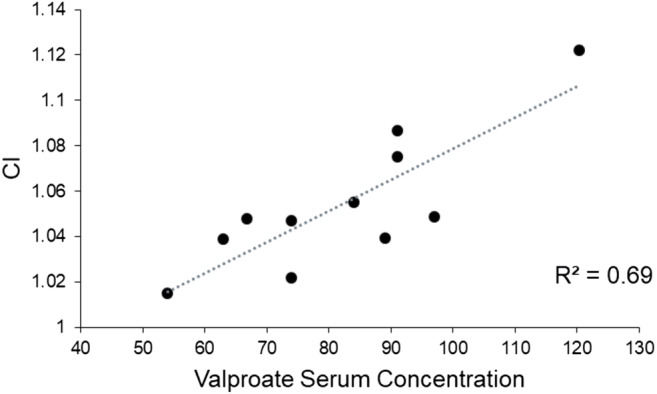
Correlation between the valproate serum concentration (μg/ml) in 11 patients with valproate-induced tremor (VIT) and curvature index (CI)

**Fig. 4:**
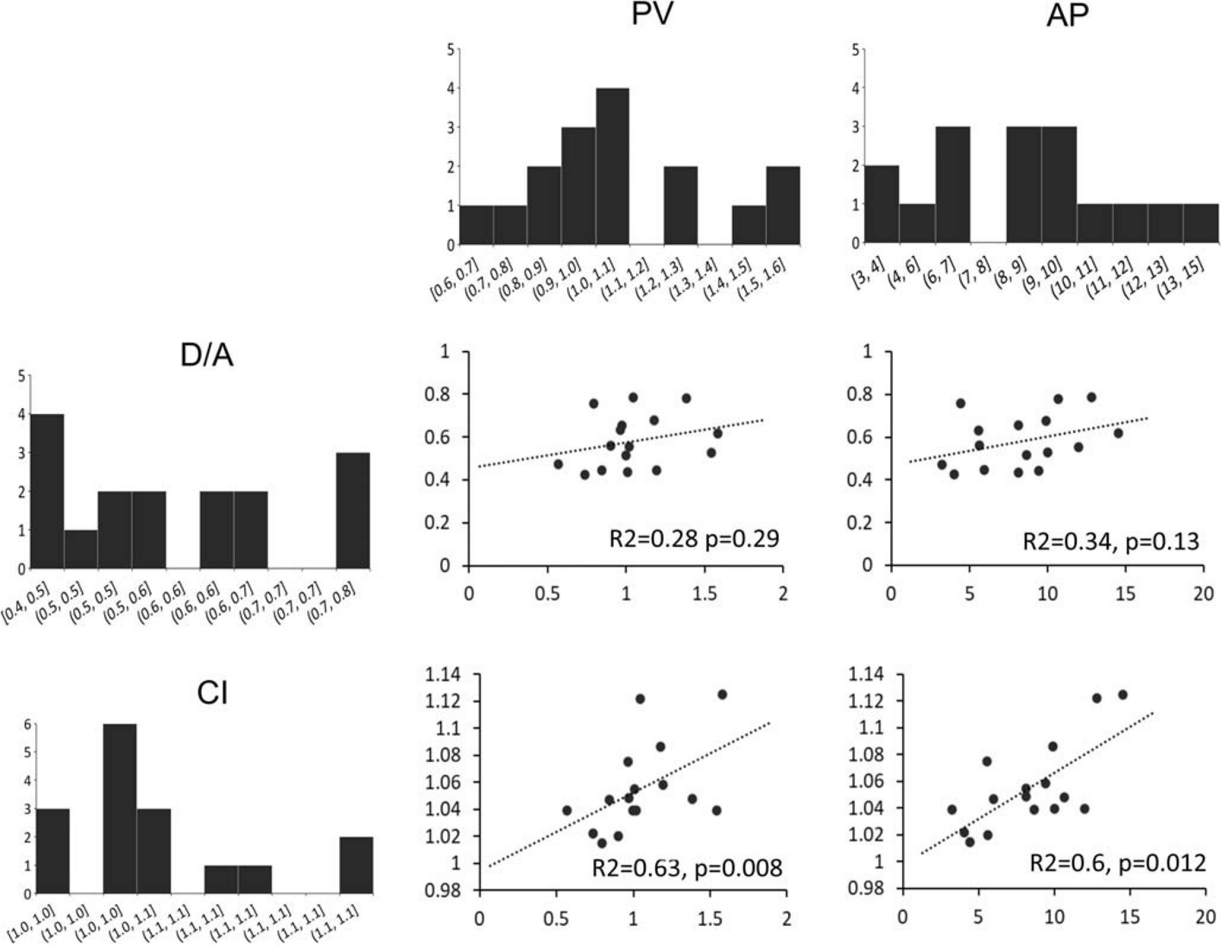
Correlation between the kinematic parameters obtained of the pointing task for the kinetic tremor assessment in patients with valproate-induced tremor (VIT). The bar charts represent on the X-axis the AP, acceleration peak, expressed in m/s^2^; the CI, curvature index; the D/A, deceleration/acceleration ratio and the PV, peak of velocity, expressed in m/s. The Y-axis represents the number of VIT patients

When we investigated possible correlations between VIT patient demographics and clinical and kinematic data, we found a positive correlation between valproate serum concentration and kinetic tremor severity, as quantified by CI during the pointing task (*R*^2^ = 0.69, *p* = 0.001 with an FDR-corrected *α* level of 0.007) (Fig. 3). When we investigated possible correlations between measures of movement execution (velocity and acceleration peaks) and movement quality (D/A and CI), we found that both velocity and acceleration peak positively correlated with CI (*R*^2^ = 0.63, *p* = 0.008 and *R*^2^ = 0.6, *p* = 0.012, with an FDR-corrected *α* level of 0.012) (Fig. 4).

### Comparisons of Clinical and Kinematic Features Between VIT and ET

Overall, VIT patients were younger than ET patients (Table 1). Mean age at tremor onset was also lower in VIT than in ET. We thus performed an additional analysis to compare VIT with an age- and sex-matched ET subgroup taken from the original ET sample, and we confirmed the results from the original patient sample (Supplementary Table 3). No differences between VIT and ET in terms of sex ratio (*p* = 0.39) or tremor duration (*p* = 0.17) emerged from the analysis. Familial history of tremor was less frequent in VIT patients than in ET patients (18.75% vs. 51.54%; *p* = 0.01). Cognitive and psychiatric scores did not differ between groups (Table 1).

As in VIT, clinical evaluation showed that all ET patients (100%) had bilateral postural and kinetic tremor of the upper limbs. However, we found a higher occurrence of cranial (head/voice) and lower limb involvement and a more frequent occurrence of rest tremor in VIT compared with ET (Table 1 and Fig. 1), thus resulting in higher FTMTRS scores in VIT (Table 1). When considering each FTMTRS subsection, VIT patients had higher scores than ET patients in sections A and B (both referring to tremor severity) and in section C (referring to tremor disability) according to the subjective perception of the patient (all *p* values < 0.05, Table 1).

**Fig. 5:**
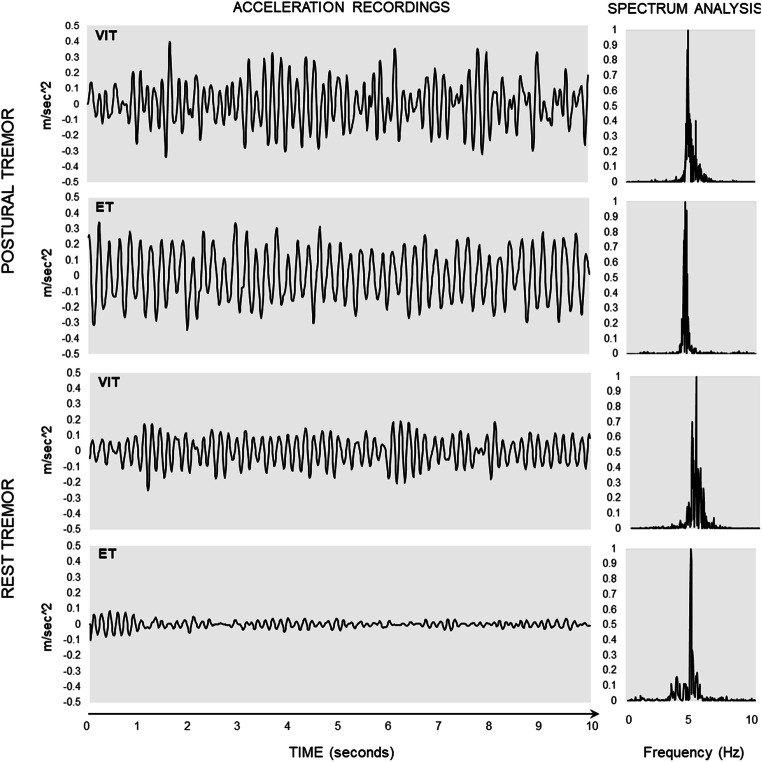
Representative kinematic traces of upper limb postural (upper part) and rest tremor (lower part) in valproate-induced tremor (VIT) and essential tremor (ET). Depicted are the acceleration recordings, expressed in m/s^2^ (left panels), and the power spectrum analysis, expressed in Hz (right panel). Note the comparable postural upper limb tremor amplitudes in VIT and ET and the higher amplitude of rest tremor in VIT compared to ET. Postural and rest tremor frequencies did not differ between VIT and ET.

When comparing the two groups in terms of kinematic data, we found comparable postural upper limb tremor features in VIT and ET (0.47 ± 0.44 vs. 0.34 ± 0.33 GRMS^2 during posture 1; 0.92 ± 1.48 vs. 0.90 ± 2.12 GRMS^2 during posture 2; 4.46 ± 1.64 vs. 4.78 ± 1.75 Hz during posture 1; 5.96 ± 1.28 vs. 5.32 ± 0.96 Hz during posture 2) (Table 3 and Fig. 5), as well as similar head tremor amplitude (0.09 ± 0.05 vs 0.14 ± 0.12 GRMS^2; 4.63 ± 1.27 vs 4.68 ± 1.20 Hz). Conversely, we found higher upper limb rest tremor amplitude in VIT than in ET (0.28 ± 0.32 vs. 0.11 ± 0.17 GRMS^2, *p* = 0.029) (Fig. 5). Although kinetic tremor severity was similar in the two groups, VIT patients performed the pointing task slower than ET patients, as demonstrated by the fewer number of movements (*p* = 0.01) and reduced peak velocity (*p* = 0.004) and acceleration (*p* = 0.002) in VIT (Fig. 2). Conversely, the duration of the acceleration and deceleration phase was longer in VIT compared with ET patients (*p* = 0.0018 and *p* = 0.0002) (Fig. 2).

## Discussion

The novel aspect of the present paper is twofold. First, we performed a clinical and kinematic characterization of VIT patients by investigating tremor in different activation conditions (i.e. postural, kinetic and rest tremor) and tremor distribution in different body segments [1]. Second, we objectively compared major tremor features in VIT with those in a large sample of patients with ET.

Results of the clinical assessment showed that VIT is characterized by bilateral postural and kinetic tremor of the upper limbs in all cases, and by the occurrence of upper limb rest tremor in a relatively high proportion of patients. With respect to previous studies, we found a relatively high occurrence of rest tremor in our sample [5, 10, 13, 14]. Another clinical observation of our study was the frequent tremor involvement of not only the upper limbs but also the cranial district (head and voice) and lower limbs, which again was higher than previously reported [2, 14]. Differences in terms of rest tremor and body distribution of tremor in VIT between our study and previous reports may be due to demographic and clinical differences, such as the younger age and shorter disease duration of VIT patients in previous reports [14] compared with our study. This issue is relevant because VIT is thought to progress over time and earlier evaluation of patients may lead to underestimation of VIT features.

In addition to clinical information, we also kinematically characterized tremor in VIT. We taped three additional markers on the trunk of participants in order to define a reference plane that allowed us to exclude possible contamination due to trunk movements from upper limb and head movement recordings [15–17]. Our methodological approach thus provided reliable results of upper limb and head tremor recordings. We found that tremor amplitude and frequency of postural, kinetic and rest upper limb tremor may considerably vary in VIT [4]. We also found a direct relationship between valproate serum concentration and kinetic tremor severity as evidenced by kinematic analysis, namely the higher CI recorded during the pointing task. Finally, our kinematic study results represent the first objective evaluation of head tremor in VIT patients. Similar to the upper limbs, kinematic analysis also showed a substantial variability in head tremor.

The second original aspect of the present study was the comparison of tremor features between VIT and ET patients. Although VIT and ET patients were all characterized by bilateral upper limb postural and kinetic tremor, we found a higher occurrence of upper limb rest tremor and the involvement of more body segments in VIT than in ET. To date, only one clinical study has compared tremor features in VIT and ET patients. That study compared tremor features in 29 VIT patients with those observed in 29 ET patients [14] and found no significant differences between the two groups. Differences in results between that study and ours may be ascribed to the sample sizes, especially considering the large clinical heterogeneity of ET [1]. Moreover, in our study, ET was diagnosed according to a recent consensus statement [1], whereas the previous study adopted earlier criteria [20]. Furthermore, we kinematically analysed tremor since this technique provides reliable results [15–17] and may also provide evidence of tremor in a larger proportion of patients than clinical examination.

Results from kinematic tremor analysis showed comparable amplitude and frequency of upper limb and head tremor in VIT and ET. These similarities may indicate that the same neural circuits are involved in the genesis of tremor in these two conditions. Again, although kinetic tremor severity was similar in the two groups, we also found that VIT patients were slower than ET patients during the pointing task. Notably, VIT patients who moved faster (higher acceleration peak) also had more severe kinetic tremor (as evidenced by higher CI values). Thus, slowed movement in VIT may represent a compensatory motor strategy in response to tremor, i.e. an attempt to minimize the detrimental effect of kinetic tremor on motor arm performance. Alternatively, slowed movement in VIT may directly reflect the detrimental effect of valproate on the cerebellar and basal ganglia function. In this regard, the cerebellum is known to contribute to the encoding of voluntary movement parameters, such as movement direction and velocity [16, 21, 22], and in line with this observation, patients with cerebellar lesions may be slow [23]. Similarly, movement slowness in VIT may be due to the involvement of basal ganglia [24, 25], which are known to play an important role in controlling movement speed [21].

The hypothesis of the detrimental effect of valproate on cerebellar function is supported by the direct relationship we found between the severity of the kinetic tremor of the upper limbs, reflected by higher CI during the pointing task, and valproate serum concentration. Accordingly, the toxic effect of valproate on the cerebellar function has been demonstrated in various studies on animals [26–30] and humans [31, 32]. Our observation of a higher occurrence of head tremor in VIT than in ET may further support the hypothesis that valproate has detrimental effects on both the cerebellar hemispheres and vermis [17, 33–35]. Finally, the relatively high occurrence of rest tremor in VIT and the toxic effects of valproate on striatal structures [3, 13, 24, 25] could suggest an involvement of basal ganglia in VIT, as observed in Parkinson’s disease patients [36–39], but the hypothesis requires further investigations.

Our results can also be interpreted with respect to the new consensus statement on tremor [1]. Accordingly, our data emphasize the importance of a detailed characterization of both activation condition and body distribution of tremor for patient classification, while tremor frequency analysis seems less relevant. Our data support the concept of largely overlapping phenomenological tremor features in different conditions. An interesting perspective that emerges from this study is the importance of considering tremor features by taking into account various activation conditions and body distributions in order to better identify distinctive elements for each aetiological condition.

We acknowledge that the present study has some limitations. First, we tested a limited sample of patients with VIT. Hence, due to the heterogeneity of tremor and epilepsy, both in terms of diagnosis and antiepileptic therapy, further studies on a larger number of participants are warranted. Moreover, future studies should focus on distinctive tremor features in focal and generalized epilepsy and in patients on different anticonvulsant therapies since other antiepileptic drugs besides valproate may affect tremor analysis (see Table 2) [2, 4]. Again, we performed only one evaluation session of VIT patients; therefore, longitudinal studies are warranted to further characterize possible changes of VIT features over time. We compared VIT patients with mild to moderate ET patients, as is demonstrated by the clinical and kinematic assessment of tremor. Hence, we cannot conclude that the results of the comparison between VIT and ET we here describe are extendable to ET patients in a more advanced stage of the disease. Finally, despite the accurate characterization of VIT through clinical and kinematic tremor assessment, physiological interpretation of the present study results does not allow us to define whether VIT is a central or enhanced physiological tremor.

In conclusion, our study provides novel information on tremor features in VIT patients and highlights relevant differences with respect to ET. Our results also provide some clues to better interpret the pathophysiological mechanisms of tremor in VIT and highlight the role of the cerebellum and basal ganglia-cerebellar interaction in this condition.

## Supplementary Information

ESM 1 (DOCX 24 kb)

## Data Availability

The data that support the findings of this study are available on request from the corresponding author (A.B).
