# Supplementary material for: Clinical and Kinematic Features of Valproate-Induced Tremor and Differences with Essential Tremor
Source: Cerebellum. 2020 Nov 16;20(3):374–83. doi: 10.1007/s12311-020-01216-5 (PMC8213593; doi:10.1007/s12311-020-01216-5)
Supplement: Supplementary file 1 — (DOCX 24 kb) [file 12311_2020_1216_MOESM1_ESM.docx]

**Supplemental Table 1: Kinematic data of postural and rest tremor recorded from the right and left upper limb and asymmetry index in VIT and ET.**

|  | **GRMS^2** | | | **Hz** | | |
| --- | --- | --- | --- | --- | --- | --- |
| **VIT** | **RUL** | **LUL** | **Asymmetry index** | **RUL** | **LUL** | **Asymmetry index** |
| **Posture 1** | **0.57 ± 0.74** | **0.36 ± 0.34** | **0.23 ± 0.23** | **4.55 ± 2.17** | **4.78 ± 1.95** | **0.14 ± 0.13** |
| **Posture 2** | **1.15 ± 2.18** | **0.68 ± 1.00** | **0.2 ± 0.18** | **5.95 ± 1.63** | **6.55 ± 1.40** | **0.08 ± 0.06** |
| **Rest tremor** | **0.28 ± 0.47** | **0.24 ± 0.39** | **0.28 ± 0.28** | **8.14 ± 2.15** | **8.03 ± 1.99** | **0.07 ± 0.04** |
| **ET** | **RUL** | **LUL** | **Asymmetry index** | **RUL** | **LUL** | **Asymmetry index** |
| **Posture 1** | **0.32 ± 0.31** | **0.36 ± 0.39** | **0.16 ± 0.17** | **4.87 ± 1.92** | **4.66 ± 2.03** | **0.14 ± 0.15** |
| **Posture 2** | **0.86 ± 1.97** | **0.92 ± 2.59** | **0.21 ± 0.19** | **4.66 ± 2.03** | **5.35 ± 1.20** | **0.07 ± 0.08** |
| **Rest tremor** | **0.14 ± 0.17** | **0.14 ± 0.21** | **0.15 ± 0.12** | **8.10 ± 2.44** | **8.13 ± 2.66** | **0.10 ± 0.09** |

**ET: essential tremor; LUL: left upper limb; RUL: right upper limb; VIT: valproate induced tremor**

**The asymmetry indexes of amplitude and frequency values recorded in VIT and ET during the posture 1, 2 and the rest position were compared by two separated repeated measures analysis of variance (rmANOVAs), with the factors GROUP (two levels: VIT vs ET) and POSTURE (three levels: P1,P2 and REST). No significant effect of the factor GROUP (F1,107=3.78; P=0.05), POSTURE (F2,214=0.41; P=0.662), nor significant interaction GROUP X POSTURE (F2,214=2.88; P=0.06) emerged from the analysis on the amplitude asymmetry index. No significant effect of the factor GROUP (F1,107=0.52; P=0.47), nor significant interaction GROUP X POSTURE (F2,214=0.43; P=0.65) emerged from the analysis on the frequency asymmetry index. The significant effect of the factor POSTURE (F2,214=4.35; P=0.01) in the rmANOVA on the frequency asymmetry index indicated that both groups presented higher frequency asymmetry index values during Posture 1.**

**Supplemental Table 2: Kinematic data of kinetic tremor recorded from the right and left upper limb and asymmetry index in VIT and ET.**

| **VIT** | | | | | **ET** | | |
| --- | --- | --- | --- | --- | --- | --- | --- |
|  | **RUL** | **LUL** | **Asymmetry index** | **RUL** | | **LUL** | **Asymmetry index** |
| **N Mov** | **7.14 ± 1.78** | **7.08 ± 1.74** | **0.05 ± 0.04** | **9.39 ± 3.83** | | **9.78 ± 3.80** | **0.11 ± 0.14** |
| **PV** | **1.02 ± 0.29** | **1.06 ± 0.28** | **0.06 ± 0.06** | **1.41 ± 0.79** | | **1.44 ± 0.75** | **0.11 ± 0.16** |
| **AP** | **8.36 ± 3.53** | **8.27 ± 3.51** | **0.09 ± 0.06** | **14.4 ± 7.76** | | **15.8 ± 11.3** | **0.12 ± 0.10** |
| **AD** | **0.71 ± 0.23** | **0.70 ± 0.21** | **0.04 ± 0.03** | **0.55 ± 0.29** | | **0.53 ± 0.31** | **0.10 ± 0.09** |
| **DD** | **0.39 ± 0.23** | **0.40 ± 0.10** | **0.08 ± 0.06** | **0.31 ± 0.14** | | **0.30 ± 0.13** | **0.07 ± 0.08** |
| **D/A** | **0.57 ± 0.15** | **0.59 ± 0.12** | **0.07 ± 0.07** | **0.64 ± 0.46** | | **0.62 ± 0.23** | **0.11 ± 0.12** |
| **CI** | **1.05 ± 0.04** | **1.06 ± 0.04** | **0.01 ± 0.01** | **1.07 ± 0.09** | | **1.04 ± 0.01** | **0.02 ± 0.08** |

**AD: acceleration duration, expressed in min; AP: acceleration peak, expressed in m/s2; CI: curvature index; D/A: deceleration/acceleration ratio; DD: acceleration duration, expressed in min; ET: essential tremor; LUL: lef upper limb; PV: peak of velocity, expressed in m/s. RUL: right upper limb; VIT: valproate induced tremor.**

**The asymmetry indexes for the kinematic parameters of the pointing task were compared by a multivariate ANOVA (MANOVA). No differences emerged between groups (Wilks lambda=0.92, F(9, 99)=0.91, P=0.51800).**

| **Supplemental Table 3** Demographic, clinical and kinematic data in VIT compared to an age-matched ET subgroup | | |  |
| --- | --- | --- | --- |
|  | **VIT (16)** | **ET (16)** | **P** |
| Gender | 7F/9M | 8F/8M | 0.5 |
| Age (years) | 46.19 ± 11.42 | 53.38 ± 13.77 | 0.121 |
| MOCA | 23.29 ± 4.43 | 25.58 ± 2.35 | 0.09 |
| FAB | 14.92 ± 2.47 | 15.87 ± 1.82 | 0.23 |
| BAI | 16.67 ± 18.23 | 15.5 ± 16.35 | 0.53 |
| BDI-II | 15.46 ± 15.58 | 14.86 ± 15.92 | 0.62 |
| Age at tremor onset (years) | 37.25 ± 17.20 | 42.03 ± 17.62 | 0.360 |
| Tremor duration (years) | 8.88 ± 8.46 | 11.47± 11.87 | 0.482 |
| Familial history | 3Y/13N | 10Y/6N | **0.015** |
| *Tremor clinical data scores* |  |  |  |
| FTMTRS tot | 27.63 ± 12.23 | 15.13 ± 7.85 | **0.003** |
| FTMTRS Section A  FTMTRS B  FTMTRS AB  FTMTRS C  MDS-UPDRS III | 10.63 ± 6 | 5.56 ± 2.42 | **0.001** |
| FTMTRS Section B | 10.19 ± 4.75 | 6.25 ± 3.96 | **0.038** |
| FTMTRS Section C | 6.81 ± 4 | 3.31 ± 2.30 | **0.01** |
| Head tremor (n of patients) | 7Y/9N (43.75%) | 4Y/12N (25%) | 0.229 |
| Voice tremor (n of patients) | 9Y/7N (56.25%) | 2Y/14N (12.5%) | **0.012** |
| Lower limbs tremor (n of patients) | 8Y/8N (50%) | 1Y/15N (6.25%) | **0.008** |
| Rest Tremor (n of patients) | 9Y/7N (56.25%) | 2Y/14N (12.5%) | **0.012** |
| *Postural and rest tremor kinematics* |  |  |  |
| UL postural tremor amplitude (GRMS^2) P1 | 0.47 ± 0.44 | 0.24 ± 0.11 | 0.05 |
| UL postural tremor amplitude (GRMS^2) P2 | 0.91 ± 1.48 | 0.35 ± 0.53 | 0.163 |
| UL postural tremor frequency (Hz) P1 | 4.46 ± 1.64 | 6.31 ± 1.98 | 0.05 |
| UL postural tremor frequency (Hz) P2 | 5.96 ± 1.28 | 5.27 ± 0.99 | 0.098 |
| UL rest tremor amplitude (GRMS^2) | 0.28 ± 0.32 | 0.11 ± 0.05 | **0.039** |
| UL rest tremor amplitude (Hz) | 8.09 ± 1.93 | 8.73 ± 2.05 | 0.368 |
| Head tremor amplitude (GRMS^2) | 0.09 ± 0.05 | 0.12 ± 0.05 | 0.199 |
| Head tremor amplitude (Hz) | 4.63 ± 1.27 | 4.28 ± 0.98 | 0.687 |
| *Kinetic tremor kinematics* |  |  |  |
| Peak Velocity (m/s) | 1.05 ± 0.28 | 1.4 ± 0.38 | **0.0046** |
| Acceleration Peak (m/s2) | 8.32 ± 3.31 | 13.3 ± 5.34 | **0.0035** |
| Acceleration Duration | 0.71 ± 0.22  2.63 | 0.51 ± 0.22 | **0.0109** |
| Deceleration Duration | 0.40 ± 0.10 | 0.31 ± 0.25 | 0.0607 |
| D/A | 0.58 ± 0.12 | 0.65 ± 0.25 | 0.2667 |
| Curvatory index (CI) | 1.05 ± 0.03 | 1.04 ± 0.02 | 0.3343 |

BAI: Beck Anxiety Inventory; BDI-II: Beck Depression Inventory; ET: Essential tremor; F: female; FAB: Frontal Assessment Battery; FTMTRS: Fahn-Tolosa-Marin Tremor Rating Scale; M: male; MOCA: Montreal Cognitive Assessment; UL.: upper limb; VIT: Valproate-induced tremor. All values are expressed as average ± standard deviation. Significant values are in bold. Note that the significant values confirmed the results from the main
